# Network Pharmacology, Molecular Docking, and Molecular Dynamics Simulation Analysis Reveal Insights into the Molecular Mechanism of *Cordia myxa* in the Treatment of Liver Cancer

**DOI:** 10.3390/biology13050315

**Published:** 2024-05-01

**Authors:** Li Li, Alaulddin Hazim Mohammed, Nazar Aziz Auda, Sarah Mohammed Saeed Alsallameh, Norah A. Albekairi, Ziyad Tariq Muhseen, Christopher J. Butch

**Affiliations:** 1 Department of Biomedical Engineering, College of Engineering and Applied Sciences, Nanjing University, Nanjing 210093, China; dg20340061@smail.nju.edu.cn; 2 School of Life Sciences and Technology, Huazhong University of Science and Technology, Wuhan 430074, China; bio_prince88@yahoo.com; 3 Department of Medical Laboratories Techniques, College of Health and Medical Techniques, Gilgamesh Ahliya University (GAU), Baghdad 10022, Iraq; nazar.azez@gau.edu.iq (N.A.A.); sarahalsallameh@gmail.com (S.M.S.A.); 4 Department of Pharmacology and Toxicology, College of Pharmacy, King Saud University, P.O. Box 2455, Riyadh 11451, Saudi Arabia; nalbekairi@ksu.edu.sa; 5 Department of Pharmacy, Al-Mustaqbal University, Hillah 51001, Iraq; 6 State Key Laboratory of Analytical Chemistry for Life Science, Jiangsu Key Laboratory of Artificial Functional Materials, Nanjing University, Nanjing 210093, China

**Keywords:** *Cordia myxa*, network pharmacology, liver cancer, traditional Chinese medicine, survival analysis, molecular docking, bioinformatics

## Abstract

**Simple Summary:**

Traditional cancer treatments have long struggled with issues such as toxicity, drug resistance, and financial burdens. However, there is growing interest in using natural compounds, like those found in complementary alternative medicine, due to their ability to influence various molecular pathways with fewer side effects. In our study, we focused on understanding how active components of *Cordia myxa* could potentially treat liver cancer (LC). By employing network pharmacology techniques, we identified key molecular targets and pathways involved. Through a combination of data analysis and computational modeling, we found that certain genes, including HSP90AA1, ESR1, CYP3A4, CDK1, and MMP9, play crucial roles in LC patient survival. Specifically, our findings suggest that compounds like cosmosiin, rosmarinic acid, quercetin, and rubinin may interact with HSP90AA1, offering a promising avenue for therapeutic intervention. Molecular dynamics simulations further validated these interactions, highlighting the stability of the drug–protein complexes. Overall, our integrated approach underscores the potential of *C. myxa* in combating LC by modulating cancer-related signaling pathways.

**Abstract:**

Traditional treatments of cancer have faced various challenges, including toxicity, medication resistance, and financial burdens. On the other hand, bioactive phytochemicals employed in complementary alternative medicine have recently gained interest due to their ability to control a wide range of molecular pathways while being less harmful. As a result, we used a network pharmacology approach to study the possible regulatory mechanisms of active constituents of *Cordia myxa* for the treatment of liver cancer (LC). Active constituents were retrieved from the IMPPAT database and the literature review, and their targets were retrieved from the STITCH and Swiss Target Prediction databases. LC-related targets were retrieved from expression datasets (GSE39791, GSE76427, GSE22058, GSE87630, and GSE112790) through gene expression omnibus (GEO). The DAVID Gene Ontology (GO) database was used to annotate target proteins, while the Kyoto Encyclopedia and Genome Database (KEGG) was used to analyze signaling pathway enrichment. STRING and Cytoscape were used to create protein–protein interaction networks (PPI), while the degree scoring algorithm of CytoHubba was used to identify hub genes. The GEPIA2 server was used for survival analysis, and PyRx was used for molecular docking analysis. Survival and network analysis revealed that five genes named heat shot protein 90 AA1 (HSP90AA1), estrogen receptor 1 (ESR1), cytochrome P450 3A4 (CYP3A4), cyclin-dependent kinase 1 (CDK1), and matrix metalloproteinase-9 (MMP9) are linked with the survival of LC patients. Finally, we conclude that four extremely active ingredients, namely cosmosiin, rosmarinic acid, quercetin, and rubinin influence the expression of HSP90AA1, which may serve as a potential therapeutic target for LC. These results were further validated by molecular dynamics simulation analysis, which predicted the complexes with highly stable dynamics. The residues of the targeted protein showed a highly stable nature except for the N-terminal domain without affecting the drug binding. An integrated network pharmacology and docking study demonstrated that *C. myxa* had a promising preventative effect on LC by working on cancer-related signaling pathways.

## 1. Introduction

Liver cancer (LC) is the fifth most common type of cancer and the third-leading cause of death globally [1]. According to the global cancer statistics reports, LC caused an estimated 781,631 deaths worldwide in 2018 [2]. According to the UK Cancer Research report, liver cancer will likely have one of the fastest rates of growth and will experience a significant rise in the number of patients by 2035 [3]. Hepatocellular carcinoma (HCC), the most prevalent type of LC, makes up between 70% and 85% of all LC [4]. HCC is strongly linked to chronic hepatitis B or C virus infection, consumption of aflatoxin-contaminated foods, and excessive intake of alcohol [5]. The majority of patients can only receive palliative care because they are typically given a diagnosis at an advanced stage and cannot undergo surgical resection. Understanding the molecular mechanisms that cause HCC and developing alternative therapies with lower toxicity levels is crucial in order to improve clinical outcomes and reduce treatment side effects [6].

*Cordia myxa* is a medicinal plant often known as “Assyrian plum and Lasura” and is a member of the “Boraginaceae” family. It is found in eastern India, tropical Africa, tropical Asia, Australia, and America [7]. Fruit extract from *C. myxa* was found to contain oil, saponins, flavonoids, glycosides, sterols, terpenoids, phenolic acids, alkaloids, coumarins, resins, gums, tannins, and mucilage [8]. It has anti-cancer, anti-bacterial, antibiotic, anti-inflammatory, hepatoprotective, anti-fungal, anti-hypertensive, anti-diabetic, anti-mitotic, and anti-oxidant properties. *C. myxa* leaves and fruit pulp have been used for centuries to treat coughs, respiratory infections, sore throats, rheumatic pain, wounds, ulcers, trypanosomiasis, skin diseases, and colic [9].

Network pharmacology (NP) in traditional Chinese medicine (TCM) is a technology that combines various fields, including computer science, systems biology, and pharmacology, which offers a unique network mode comprising “multiple targets, multiple effects, and complicated diseases” [10]. It associates drugs and diseases in a broader sense, and it provides different approaches for investigating the mechanisms of traditional Chinese medicine by introducing and developing new drugs [11]. Bioinformatics is an innovative field that integrates molecular biology with mathematics, statistics, computer science, and other disciplines. It can be used to examine the relationships and laws that govern biological genes and diseases. Furthermore, it has rapidly evolved into the most appealing frontier of life sciences nowadays [12]. Batool et al. [13] employed both bioinformatics and network pharmacology to elucidate the anti-cancer effect of Fumaria indica to treat liver cancer. Sadaqat et al. [14] implemented an advanced network-pharmacology-based approach to examine the active components of *Bacopa monnieri* for the treatment of liver cancer.

The present study utilized a network pharmacology approach to investigate the active ingredients and potential targets of *C. myxa* for liver cancer treatment. This approach constructs models that consider multiple components and targets, providing a comprehensive understanding of the complex interactions between active compounds and target proteins. In addition, survival analysis and molecular docking studies were conducted to validate the results. Furthermore, the obtained results were supplemented by all-atom molecular dynamics (MD) simulation for 100 ns, followed by MMGBA/PBSA analysis to examine the conformational changes, stability, and interaction mechanism of target proteins when bound to the proposed compounds. This study is the first to explore the efficacy and mechanism of *C. myxa* in liver cancer treatment, offering theoretical support and guidance for future research. It provides valuable insights into the molecular mechanisms underlying the anti-liver-cancer activity of *C. myxa* and accelerates the drug discovery process. However, further wet lab experiments are required to analyze the pharmacological potential of *C. myxa*-related compounds.

## 2. Materials and Methods

### 2.1. Collection and Screening of Active Constituents and Corresponding Targets

Active compounds of *C. myxa* were obtained from the review of the literature and the Indian Medicinal Plants, Phytochemistry, and Therapeutics database (IMPPAT; https://cb.imsc.res.in/imppat/, accessed on 29 December 2022) [15]. Using canonical smiles, the bioactive compounds were obtained using the oral bioavailability (OB) ≥ 30% and drug-likeness (DL) ≥ 0.18 retrieval filters through SwissADME (http://www.swissadme.ch/, accessed on 1 January 2023) [16] and Molsoft (https://molsoft.com/mprop/, accessed on 1 January 2023) [17], respectively. The amount and pace at which oral medicine is absorbed into the systemic circulation are referred to as OB [18]. DL is a chemical qualitative characteristic that is commonly used during the early phases of drug discovery [19]. The compound ID, canonical smiles, and molecular weight (MW) were retrieved from the PubChem (https://pubchem.ncbi.nlm.nih.gov/, accessed on 2 January 2023) database [20]. The 2D structures of active constituents were drawn through the RDKit package of Python [21].

The potential targets related to active constituents of *C. myxa* were investigated and evaluated using public databases such as the Swiss Target Prediction (http://www.swisstargetprediction.ch/, accessed on 5 January 2023) [22] and STITCH (http://stitch.embl.de/, accessed on 5 January 2023) databases [23]. Once the target was predicted, the species in each of these databases was confined to *Homo Sapiens*. The active constituent–target network was constructed using Cytoscape version 3.9.1 [24].

### 2.2. Identification of Critical Genes in LC from Expression Datasets

Five microarray datasets (GSE39791, GSE76427, GSE22058, GSE87630, and GSE112790) were selected for the identification of differentially expressed genes (DEGs) in LC. The NCBI-GEO database (https://www.ncbi.nlm.nih.gov/geo/, accessed on 6 January 2023) [25] was used to retrieve these datasets. GSE39791, GSE76427, GSE22058, GSE87630, and GSE112790 consisted of 144 (72 normal and 72 affected), 167 (52 normal and 115 affected), 197 (97 normal and 100 affected), 94 (30 normal and 64 affected), and 198 (15 normal and 183 affected) tissue samples. Limma v.3.26.8 package of R language was used for the normalization of datasets from which data redundancy is eliminated, and data alteration errors are minimized [26]. The genes having adjusted *p*-value < 0.05, log (FC) < −1, and log (FC) > 1 were considered significant DEGs and defined as LC-specific genes. To illustrate major up-regulated and down-regulated genes, volcano plots were created for the LC vs. normal comparison. The DEGs acquired from the preceding technique were used for further analysis. Targets from the compound target databases and GEO datasets (non-redundant) were combined, and a Venn diagram was constructed to highlight the genes shared by *C. myxa* and LC targets.

### 2.3. Pathways and Gene Ontology (GO) Enrichment Analysis of Potential Targets

Gene Ontology (GO) analysis is currently a popular way to analyze genomic data, particularly large-scale transcriptome data. Potential targets were analyzed for GO functional enrichment in 3 groups: biological process (BP), cellular component (CC), and molecular function (MF). The DAVID database (https://david.ncifcrf.gov/home.jsp/, accessed on 8 January 2023) [27] was used to perform GO functional and KEGG pathway enrichment analyses on target genes. The enriched GO keywords and pathways with *p*-values less than 0.05 were chosen for visualization. A package of R “ggplot2” was used to create a bubble graph of the top 20 significant pathways and GO terms (BP, CC, and MF) [28].

### 2.4. Protein–Protein Interactions (PPIs) and Network Analyses

PPI is the process through which two or more protein molecules form protein complexes via noncovalent bonding. The STRING database (https://string-db.org/, accessed on 9 January 2023) [29] was used to assess the relationship between LC therapeutic targets. *Homo sapiens* was selected as the reference organism, and the total score was set to 0.5 or higher. The PPI network was visualized using Cytoscape, and the CytoHubba plugin was utilized to find hub genes based on the degree method and higher-degree nodes [30].

A network of active constituents–targets–pathways was generated using Cytoscape software, version 3.9.1, to characterize the therapeutic mechanisms of *C. myxa* for LC. The nodes with different colors and geometries in the network represent active constituents, target genes, and pathways, respectively, and an “edge” represents a link between the nodes.

### 2.5. Survival Analysis

To investigate the impact of the hub targets on the overall survival (OS) of LC, a cancer genomics server called GEPIA 2 (http://gepia2.cancer-pku.cn/#index, accessed on 12 January 2023) [31] was used to quantify the prognostic importance of each hub gene. A Kaplan–Meier survival plot was used to compare the two groups of LC patients who were classified into high- and low-expression groups [32]. Hazard ratios (HRs; 95% confidence intervals) and logrank *p* values were determined for survival, with logrank *p* < 0.05 serving as the statistical significance threshold [33].

### 2.6. Molecular Docking

The Protein Data Bank (PDB; https://www.rcsb.org/, accessed on 13 January 2023) [34] was used to search and download the target protein structure predicted by the X-ray crystallography method. UCSF Chimera was used for the removal of non-standard atoms, solvents, and energy minimization [35]. The online tool Computed Atlas of Surface Topography of Protein (CASTp; http://sts.bioe.uic.edu/castp/index.html?2cpk, accessed on 13 January 2023) [36] was used to predict binding pockets of target protein. The PubChem database (https://pubchem.ncbi.nlm.nih.gov/, accessed on 2 January 2023) [20] was used to download 3D structures of compounds. PyRx software v0.8 was used for virtual screening and molecular docking of target protein with drug molecules [37]. Two- and three-dimensional interactions of docking complexes were visualized using Discovery Studio [38] and ChimeraX [39], respectively.

### 2.7. Analysis of Molecular Dynamic Simulation

The molecular dynamic simulation was performed using the AMBER22 program [40]. An in silico simulation method called molecular dynamic simulations (MDs) is mostly used to understand intermolecular dynamics along the simulation time [41]. In a molecular dynamic simulation pipeline, the atom and molecular trajectories are generated by solving Newton’s equations of motion, and a macromolecule is permitted to exhibit dynamic behavior for a predetermined period of time [42]. In this study, a 100 ns computer simulation was utilized to assess the drugs’ dynamic behavior using the AMBER22 program [43]. The antechamber program was used to prepare the systems, and GAFF2 and FF19Sb were employed as force fields for parameterizing the complexes. This was done to read how the ligands’ drug affinities for the receptor gene changed over time. To attain charge neutrality, the right number of counter ions was added to the system [44]. A cubic box of OPC with a size of eight angstroms was considered sufficient to solvate the complexes. Energy minimization of the complexes was carried out through steepest descent and conjugate gradient. The complexes were heated to 310 K for 500 ps, followed by equilibration and production run for 100 ns [45]. The temperature during the production run was maintained through the Langevin dynamics algorithm, while the hydrogen bonded atoms were constrained via SHAKE algorithm. The generated trajectories were structurally investigated via the CCPTRAJ module [46].

### 2.8. MMPB/GBSA Analysis

MMPB/GBSA analysis was used to predict the binding free energies of docked ligands with the HSP90AA1 gene [47,48]. A script from the AMBER v22 program named MMPBSA.py was used to accomplish this [49]. The script took into account 5000 frames from the paths that were chosen at regular intervals. The MMPB/GBSA energy formula is as follows:

∆Gbinding = Gcomplex − (Gprotein − ∆Gligand).

**Figure 1:**
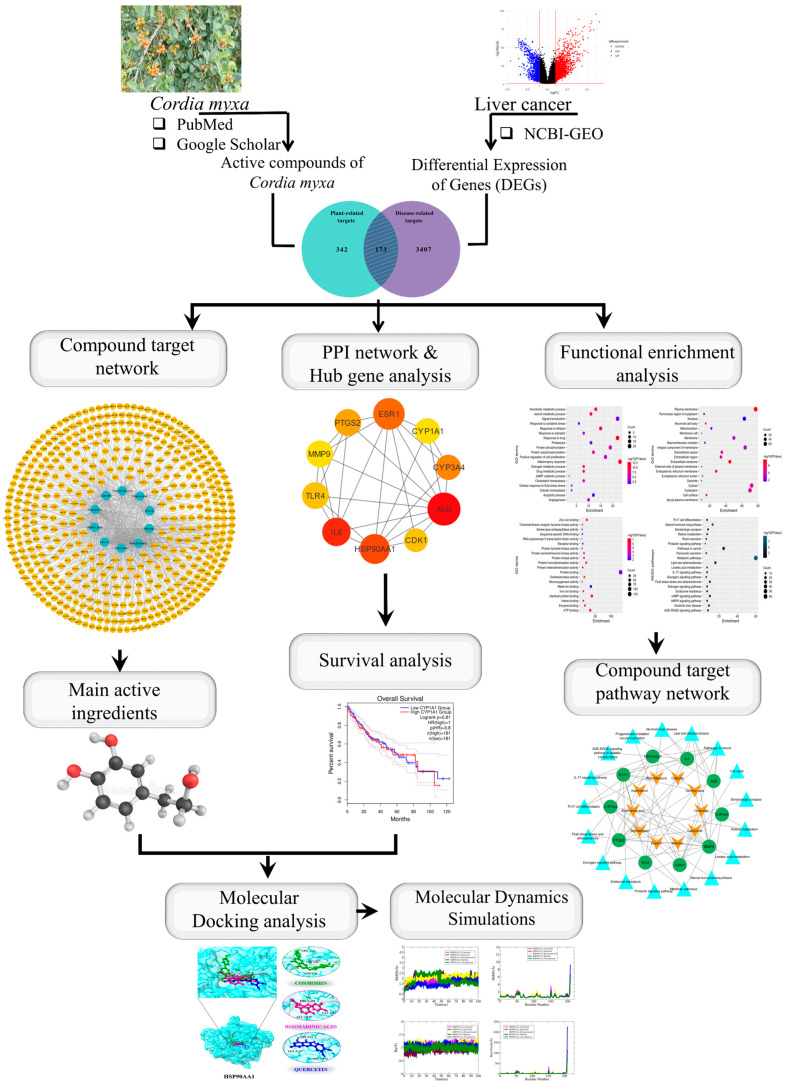
Graphical representation of the workflow of this study.

The free ligand energy is represented by ΔGligand, the free energy of the receptor protein by ΔGreceptor, the complex free energy by ΔGcomplex, and the overall binding free energy by ΔGbind. To determine the distinct free energies of a complex, protein, and ligand, utilize the following formula. The results show that the MM/PBSA and MM/GBSA methods function similarly. The MM/GBSA uses the Generalized Born equation, which is thought to be quicker to solve the previous equation, for determining the electrostatic energy contribution to the free energy, whereas the MM/PBSA uses the Poisson–Boltzmann equation [40]. Figure 1 illustrates the whole methodology used in this study.

## 3. Results

### 3.1. Identification and Filtration of Active Constituents of C. myxa

After searching, identification, screening, and removal of duplications, a total of 10 putative compounds including allantoin, beta-sitosterol, cosmosiin, catechin, gentisic acid, kaempferol, quercetin, rosmarinic acid, rubinin, and stigmastanol with OB ≥ 30% and DL ≥ 0.18 were selected as novel compounds (Table 1) [50].

### 3.2. Identification and Screening of Potential Targets for C. myxa and LC

**Figure 2:**
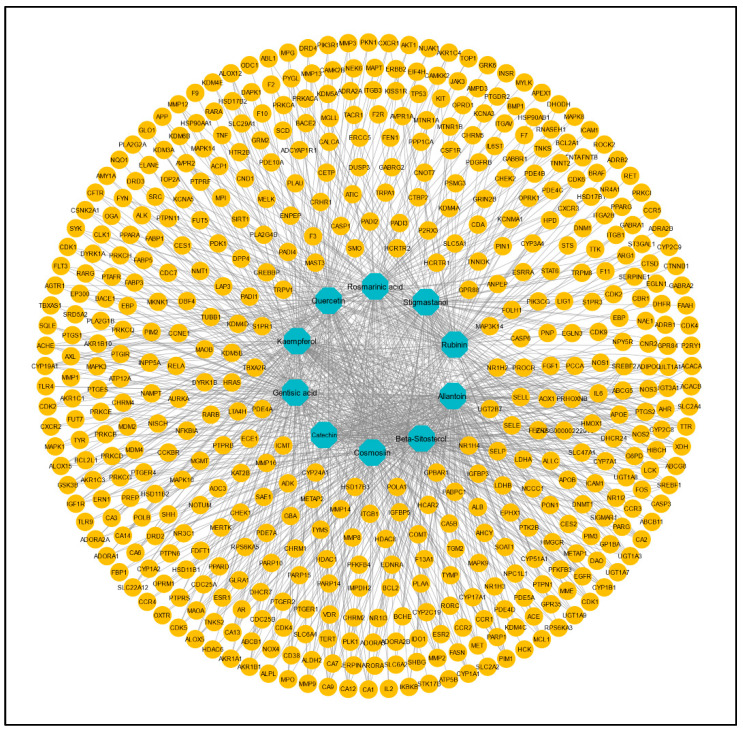
Compound–target network of 173 common targets of 10 active compounds. Blue color represents the compounds, and yellow color represents the targets.

From these 10 active constituents, 515 potential target genes were retrieved from the Swiss Target Prediction and STITCH databases. An active constituent–target network was constructed using Cytoscape version 3.9.1. There were 525 nodes and 1057 edges in the network (Figure 2). The dark-cyan nodes represent active compounds, while the orange nodes represent targets. The CytoHubba plugin of Cytoscape was used to calculate the degree and other parameters (MNC, MCC, closeness, betweenness) of active constituents (Table 2).

**Figure 3:**
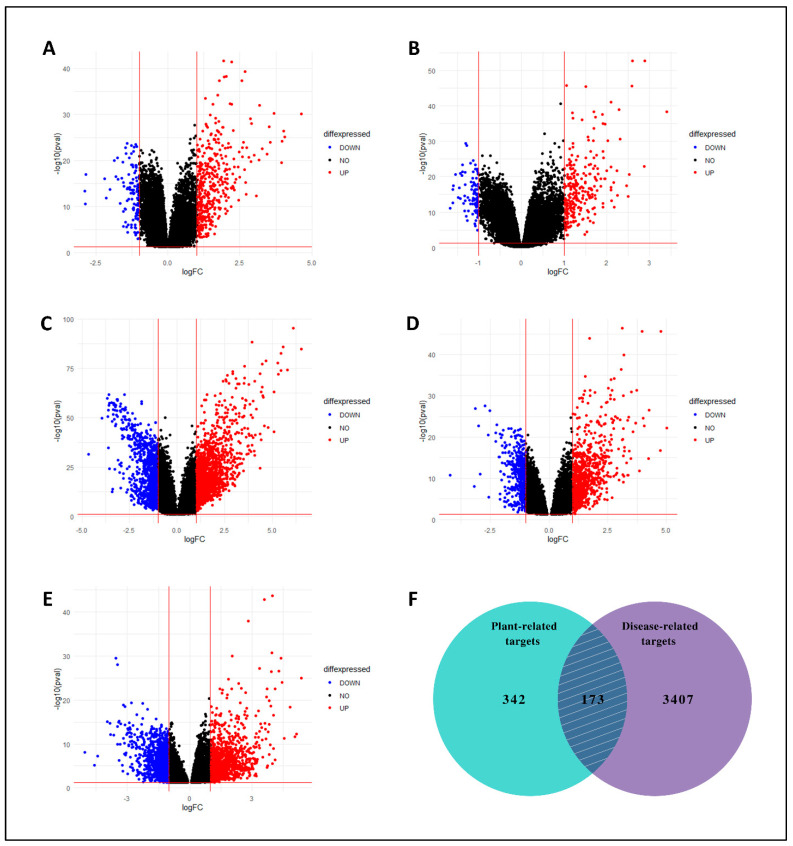
Volcano plots of DEGs. (**A**) GSE39791, (**B**) GSE76427, (**C**) GSE22058, (**D**) GSE87630, and (**E**) GSE112790. Blue and red colors represent down-regulated and up-regulated genes, respectively. (**F**) With a total of 173 overlapping genes, the Venn diagram shows the targets of *C. myxa* and LC.

On the other hand, from five GEO expression datasets, collectively 3580 DEGs related to LC were identified between LC and normal tissues. The volcano plots were generated using the DEGs from all five datasets (Figure 3A–E). Both compound-related and LC-related target genes were submitted to find overlapped/mutual genes through the Venn diagram, and 173 mutual targets were obtained (Figure 3F). These targets were assumed as key targets and proceeded for further analysis.

### 3.3. Pathways and GO Enrichment Analysis

**Figure 4:**
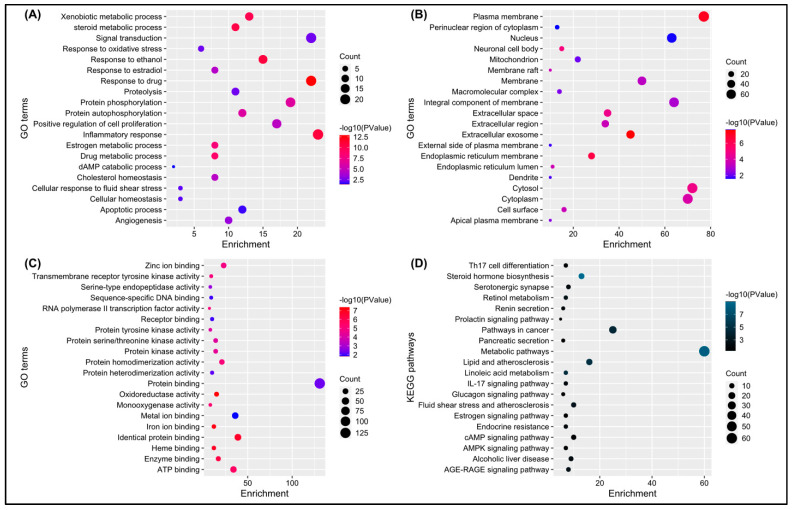
(**A**–**D**) depicts a bubble chart of the top 20 enriched GO terms (BP, CC, MF) and KEGG pathways, respectively.

The DAVID database provided a total of 204 significant biological processes, 49 cellular components, 92 molecular functions, and 46 KEGG pathways terms. According to the biological processes (BPs), the target genes are mainly involved in response to the drug, inflammatory response, response to ethanol, and so forth (Figure 4A). Cellular components (CCs) indicate that most of the genes are present in the plasma membrane, extracellular exome, cytosol, and so forth (Figure 4B). Molecular functions revealed that genes are involved in protein, ATP, zinc ion binding, and so forth (Figure 4C). KEGG pathway analysis showed that genes are mainly involved in metabolic pathways, pathways in cancer, steroid hormone biosynthesis, and so forth (Figure 4D).

### 3.4. Interaction of Protein with Other Proteins (PPI)

**Figure 5:**
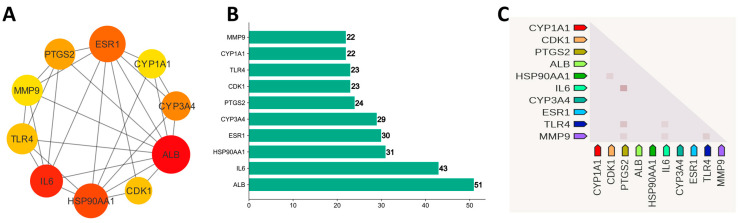
(**A**) Ten hub genes based on degree. (**B**) Bar chart of 10 hub genes. (**C**) Co-expression of hub genes in Homo sapiens.

Using STRING version 11.5, the 173 potential genes were linked to form an initial PPI network. The output file was downloaded in tsv format, and a filter on a combined score ≥ 0.5 was applied. The file was taken as input into the Cytoscape version 3.9.1 to construct and check the significant interactions among proteins in a pharmacological network. There were 161 nodes and 725 edges in the network. The degree scoring algorithm of CytoHubba was applied to the network to find the top 10 hub genes (Figure 5A,B). ALB (51), IL6 (43), HSP90AA1 (31), ESR1 (30), CYP3A4 (29), PTGS2 (24), TLR4 (23), CDK1 (23), MMP9 (22), and CYP1A1 (22) have the higher degree and proceed further for drug–target–pathways network and survival analysis. Figure 5C shows the co-expression relationships of hub genes among each other. The dark color indicates high confidence in relationships [51].

### 3.5. Construction of the Drug–Target–Pathways Network

**Figure 6:**
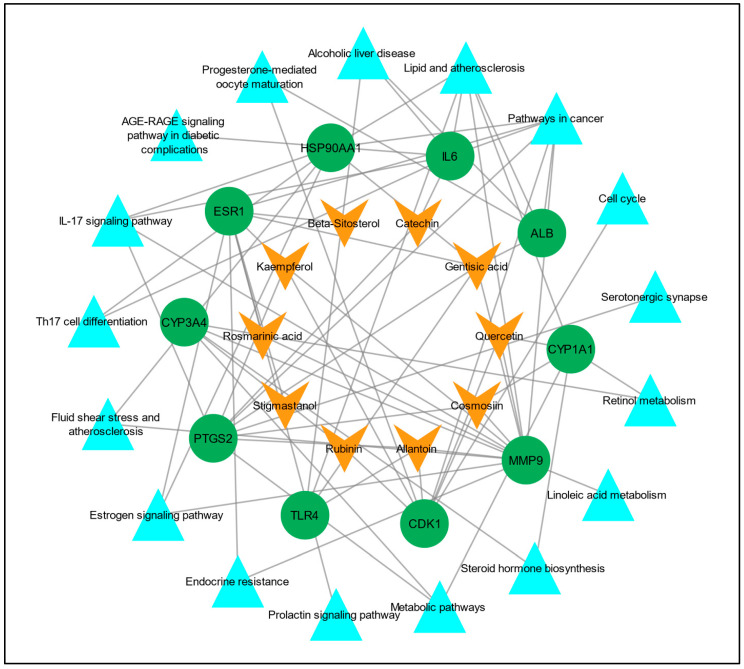
Compound/drug–target–pathways network. The orange V shapes represent the active constituents/drugs associated with hub genes, the green circles represent hub genes, and the cyan triangles represent the pathways linked to the hub genes.

To understand the multi-target effect of *C. myxa* in LC, two networks, the “drug–target network” and “target–pathways network” were constructed separately in Cytoscape version 3.9.1. In the drug–target network, there were 20 nodes and 30 edges, and in the target–pathways network, there were 27 nodes and 43 edges. Later, these two networks were merged to construct a drug–target–pathways network, and there were 37 nodes and 72 edges in the merged network (Figure 6).

### 3.6. Survival Analysis

**Figure 7:**
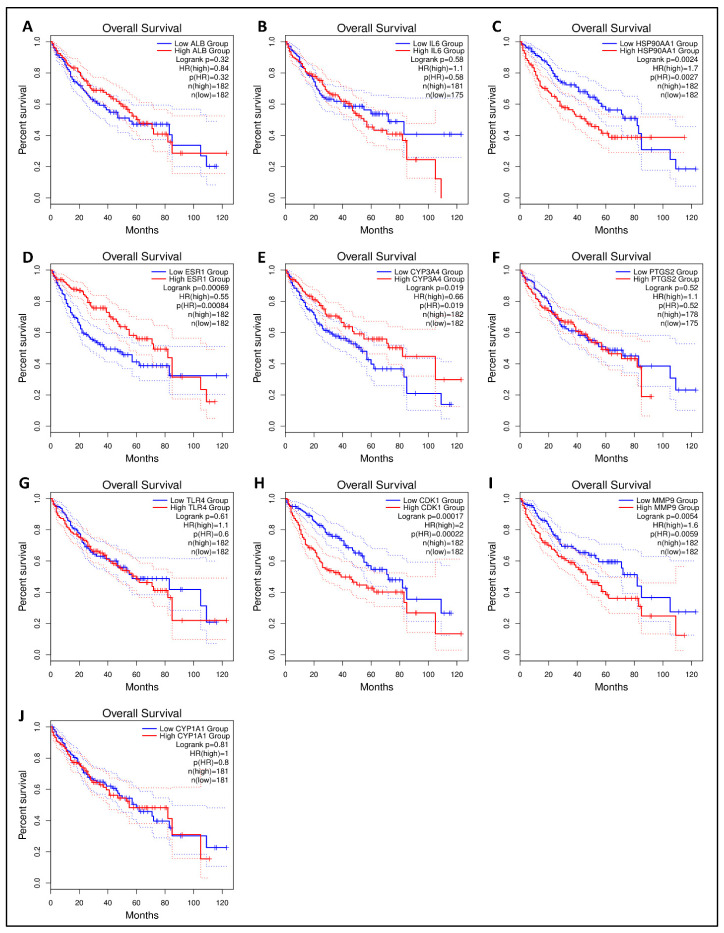
The GEPIA 2 was used to evaluate the survival data of the hub genes including (**A**) ALB, (**B**) IL6, (**C**) HSP90AA1, (**D**) ESR1, (**E**) CYP3A4, (**F**) PTGS2, (**G**) TLR4, (**H**) CDK1, (**I**) MMP9, and (**J**) CYP1A1. The red line shows patients with expression levels above the median, whereas the black line reflects expression levels below the median. HR stands for the hazard ratio.

The Kaplan–Meier survival plot was used to examine the disease-free survival of the hub genes in LC to further investigate if hub genes contributed to the prognosis in patients [52]. From 10 hub genes, HSP90AA1, ESR1, CYP3A4, CDK1, and MMP9 were linked to overall survival in all LC patients (logrank *p* < 0.05), suggesting that they may prevent LC development. There was no statistical significance (logrank *p* < 0.05) in the overall survival analysis of the remaining five core genes with high and low expression (Figure 7). One hub gene (HSP90AA1) having a higher degree and significance in survival was proceeded further for molecular docking analysis.

### 3.7. Molecular Docking

**Figure 8:**
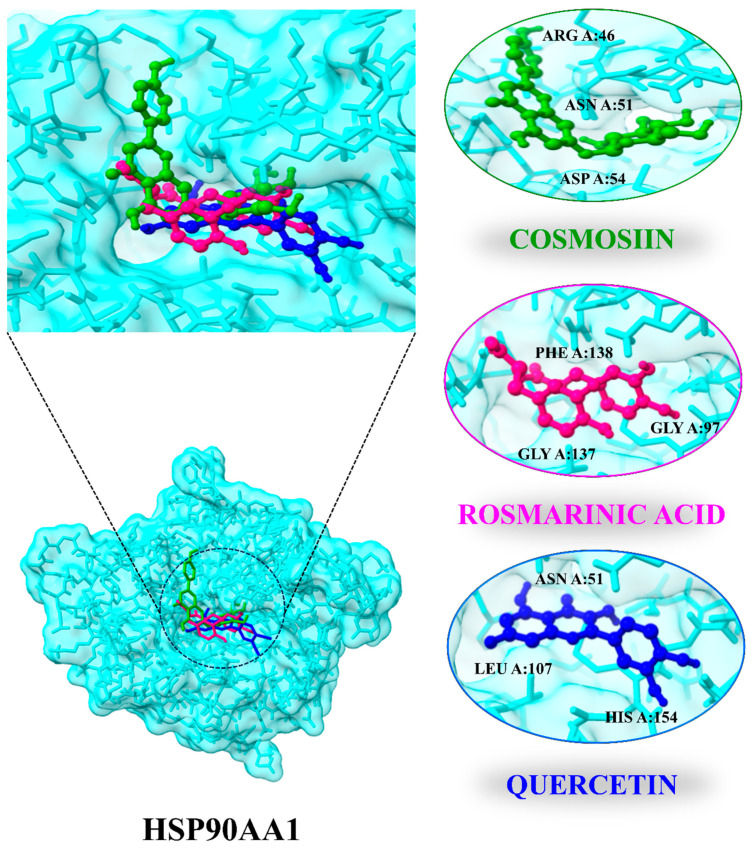
Docking position and interactions of 3 highly bounded compounds with HSP90AA1.

Ten compounds were docked with the HSP90AA1 (PDB ID: 4BQG) target protein in this experiment. All compounds demonstrated good binding and a high degree of matching with the target protein. Moreover, alvespimycin [53,54] was identified as a positive control drug of HSP90AA1. The results demonstrated that HSP90AA1 has a higher binding affinity with cosmosiin (−7.3 kJ mol), rosmarinic acid (−7.2 kJ/mol), quercetin (−6.7 kJ /mol), and rubinin (−6.7 kJ/mol) compared to alvespimycin (−6.0 kJ/mol). Cosmosiin and rosmarinic acid side chains form hydrogen bonds with ARG A:46, ASN A:51, and ASP A:54 and with LYA A:58, GLY A:97, MET A:98, and GLY A:137, respectively, while quercetin side chains form hydrogen bonds with ASN A:51, GLY A:97, NET A:98, LEU A:107, and HIS A:154. Rubinin side chains also form stable bonds with ASN A:51, ALA A:55, LYS A:58, MET, and A:98 residues. As a result, these findings suggest that active *C. myxa* components bind stably to the HSP90AA1 target protein and serve as an LC repressor. Additionally, subsequent research will concentrate on the active ingredients binding pockets with the core protein (Figure 8; Table 3). In comparison with control drug, all compounds show stable binding with ASN A:51 residue except rosmarinic acid. The RMSD of cosmosiin (1.183 Å), rosmarinic acid (2.552 Å), and quercetin (1.136 Å) is lower compared to alvespimycin (2.631 Å) suggesting these have potential to effectively bind with the binding pocket of the target protein.

### 3.8. Molecular Dynamic Simulation

Molecular dynamic simulation studies essentially validate the dynamic behavior of macromolecules. Radius of gyration (RoG), root mean square fluctuation (RMSF), and root mean square deviation (RMSD) are all included in the simulations analysis. The carbon alpha atom of the complexes served as the basis for all of these investigations. These studies sought to determine whether interactions between the ligand and receptor persisted during the simulation period and whether the binding was stable. Ensuring that the ligand is correctly delivered to the HSP90AA1 target protein is dependent on stable receptor–ligand interaction. There were no obvious structural alterations at first, as seen by the systems’ uniform RMSD plot.

**Figure 9:**
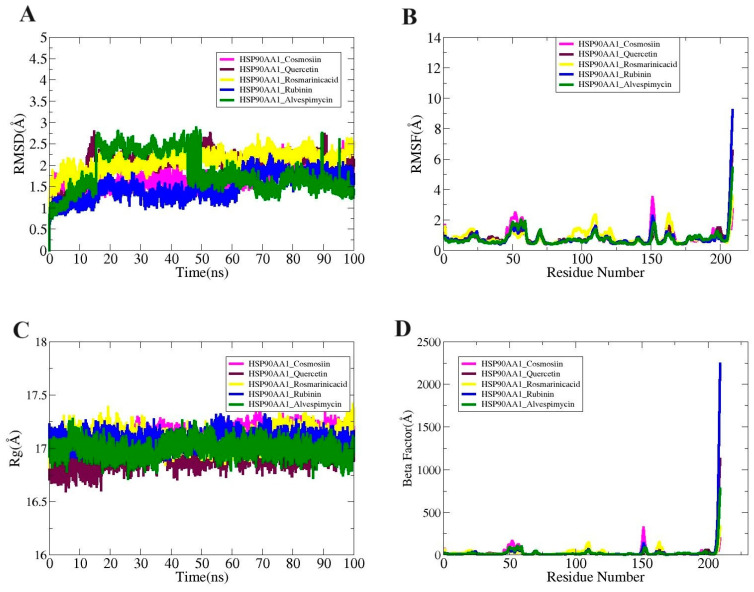
(**A**) RMSD, (**B**) RMSF, (**C**) RoG, and (**D**) Beta Factor plots for the complexes.

While the greatest values of the systems’ root mean square deviation (RMSD) ranged <3 Å, the mean values of HSP90AA1_Cosmosiin, HSP90AA1_Quercetin, HSP90AA1_Rosmarinic acid, HSP90AA1_ Rubinin, and HSP90AA1_Alvespimycin were determined to be 1.71 Å, 2.02 Å, 2.08 Å,1.49 Å, and 1.81 Å, respectively (Figure 9A). Secondly, the RMSF was computed to disclose details regarding the adaptability of the receptor residues when the ligand molecule is present (Figure 9B). The majority of system residues fell under the average stability range (<5 Å). The root mean square fluctuation (RMSF) for HSP90AA1_Cosmosiin, HSP90AA1_Quercetin, HSP90AA1_Rosmarinic acid, HSP90AA1_ Rubinin, and HSP90AA1_Alvespimycin was 3.48Å, 6.54Å, 3.55 Å, 9.12 Å, and 5.87 Å at the maximum; 0.80Å, 0.84 Å, 0.86 Å, 0.67 Å, and 0.60 Å at the mean; and 0.40 Å, 0.40 Å, 0.43 Å, 0.31 Å, and 0.39 Å at the lowest value. It was demonstrated that the loop pressure within the system was the cause of the greater degree of flexibility observed in certain of the residues. Using the RoG analysis, the system’s compactness was examined over time. These differences did not impact the manner in which ligands bound to the receptors, however. The RoG maximum values of 17.36 Å, 17.21 Å, 17.41 Å, 17.31 Å, and 17.27 Å; mean values of 17.09 Å, 16.90 Å, 17.09 Å, 17.08 Å, and 16.99 Å; and minimum values of 16.75 Å, 16.59 Å, 16.82 Å, 16.88 Å, and 16.71 Å were observed for HSP90AA1_Cosmosiin, HSP90AA1_Quercetin, HSP90AA1_Rosmarinic acid, HSP90AA1_ Rubinin, and HSP90AA1_Alvespimycin, respectively (Figure 9C). By the end of the simulation, RMSD showed that every system was compact and had not undergone any significant changes. The following findings were obtained from the Beta Factor study: According to Figure 9D, the mean values of HSP90AA1_Cosmosiin, HSP90AA1_Quercetin, HSP90AA1_Rosmarinic acid, HSP90AA1_Rubinin, and HSP90AA1_Alvespimycin were 23.02 Å, 28.05 Å, 24.58 Å, 35.53 Å, and 23.19 Å; the lowest values were 4.24 Å, 4.27 Å, 4.94 Å, 4.21 Å, and 3.95 Å; and the maximum values were 320.20 Å, 1128.2 Å, and 33.3 Å, 2239.78 Å, and 776.08 Å.

### 3.9. Solvent-Accessible Surface Area Analysis

**Figure 10:**
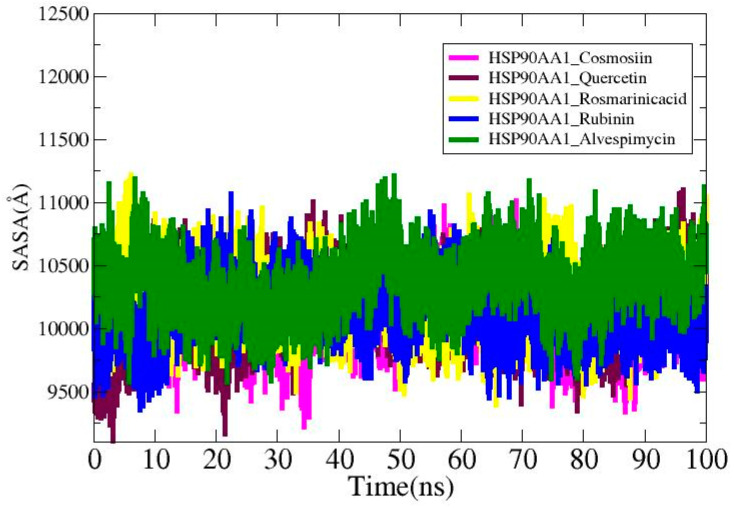
SASA analysis for the studied complexes.

Solvent-accessible surface area (SASA) study was carried out for the ligands in order to find out more about the surface area of the HSP90AA1 that interacts with the solvent molecules. The average values for the systems are HSP90AA1_Cosmosiin (10,096.2 Å^2^), HSP90AA1_Quercetin (10,143.4 Å^2^), HSP90AA1_Rosmarinic acid (10,249.5 Å^2^), HSP90AA1_ Rubinin (10,135.2 Å^2^), and HSP90AA1_Alvespimycin (10,357.6 Å^2^). The lowest SASA values for HSP90AA1_Cosmosiin, HSP90AA1_Quercetin, HSP90AA1_Rosmarinic acid, HSP90AA1_ Rubinin, and HSP90AA1_Alvespimycin were 9221.03 Å^2^, 9107.7 Å^2^, 9397.7 Å^2^, 9358.71 Å^2^, and 9576.98 Å^2^, as shown in Figure 10. The highest values recorded were 11,016 Å^2^, 11,103.4 Å^2^, 11,218.4 Å^2^, 11,068 Å^2^, and 11,208 Å^2^ in that order. Plots display the notable differences that are seen upon ligand binding.

### 3.10. MMPB/GBSA Analysis

The docked complexes that were selected underwent an investigation using the MMPB/GBSA method. These techniques are considered more successful in determining the binding affinity between the docked ligand and the receptor protein. The formation of strong intermolecular systems and stable complexes is evident from the highly negative net binding energies observed in all the docked and control complexes. The dominant force responsible for the stability of the complexes is the van der Waals force, which ensures the docking of the ligands at the designated site and stabilizes the systems. Specifically, the net van der Waals energies of HSP90AA1_Cosmosiin, HSP90AA1_Quercetin, HSP90AA1_Rosmarinic acid, HSP90AA1_ Rubinin, and HSP90AA1_Alvespimycin were found to be −41.25 kcal/mol, −46.01 kcal/mol, −44.69 kcal/mol, −51.01 kcal/mol, and −55.94 kcal/mol, respectively.

Furthermore, the electrostatic energies of each docked complex were remarkably consistent. Among all the computed energies, the solvation energy had the least impact and made a negative contribution to the net energy. In MM-GBSA, the net solvation energies for HSP90AA1_Cosmosiin, HSP90AA1_Quercetin, HSP90AA1_Rosmarinic acid, HSP90AA1_Rubinin, and HSP90AA1_Alvespimycin were 8.08 kcal/mol, 7.14 kcal/mol, 7.78 kcal/mol, 8.66 kcal/mol, and 8.09 kcal/mol, respectively. On the other hand, in MM/PBSA, the net solvation energies for the same compounds were 7.52 kcal/mol, 8.06 kcal/mol, 7.65 kcal/mol, 8.16 kcal/mol, and 8.04 kcal/mol, respectively. Additional details regarding the energy terms and their corresponding values can be found in Table 4.

## 4. Discussion

Disease treatments with many components and different targets have received increased interest, and this is one of the benefits of traditional Chinese medicine. *C. myxa* has traditionally been used in folk medicine. Many of its constituents have been demonstrated in studies to have anti-cancer properties and can help prevent the development of LC [55]. However, the precise processes of *C. myxa* in LC therapy have not yet been fully established. The active components of *C. myxa* and the mechanisms linked with the therapeutic effect of *C. myxa* on LC were investigated using network pharmacology, survival analysis, and molecular docking.

In the current investigation, the active components and associated targets of *C. myxa* were screened from the IMPPAT, Swiss Target Prediction, and STITCH databases. DEGs were extracted from LC datasets GSE39791, GSE76427, GSE22058, GSE87630, and GSE112790 using the *p*-value < 0.05 and logFC > 1 for up- and logFC < −1 for down-regulated genes [56]. Plant-related and disease-related DEGs were intersected to find overlapping targets, and 173 potential target genes were found. Using the degree threshold, 10 hub genes were identified from the PPI network.

The GO functional enrichment study revealed that the hub genes were related with GO keywords such as drug response, inflammatory response, ethanol response, positive regulation of cell proliferation, plasma membrane, cytosol, endoplasmic reticulum membrane, extracellular exosome, identical protein binding, protein heterodimerization activity, and binding of zinc ion. The KEGG pathways related to the hub genes include metabolic pathways, cAMP signaling pathway, cancer pathway, alcoholic liver disease, AGE-RAGE signaling pathway in diabetic complications, IL-17 signaling pathway, and AMPK signaling pathway. After a survival analysis, and drug–target–pathway analysis, five hub genes (HSP90AA1, ESR1, CYP3A4, CDK1, and MMP9) were discovered to be involved in the overall survival of LC patients. These five genes have been identified as targets of *C. myxa’s* active constituents associated with LC, making them the most dependable genes for use in clinical studies.

The results of the network analysis and survival analysis indicated HSP90AA1 as an important protective factor in LC treatment. Higher HSP90AA1 expression is linked to depression in HCC patients [57]. From survival and network analysis, one essential target HSP90AA1 was tested for anti-LC effectiveness by binding with ten active components of *C. myxa*. The docking analysis results confirmed our findings, which revealed that the chemicals cosmosiin, rosmarinic acid, quercetin, and rubinin can have stable interactions with the binding sites of the target gene. The binding affinity and RMSD value indicate that the active constituents of *C. myxa* have higher binding affinity and lower RMSD compared to the positive control drug alvespimycin, which indicates that these constituents have more potential and stable binding with HSP90AA1.

Animal models play a crucial role in understanding the role of HSP90AA1 in liver cancer. Research has shown that targeting HSP90AA1 can lead to inhibition of cancer cell proliferation and survival, making it a potential therapeutic target [57,58]. These animal models allow researchers to study the effects of HSP90AA1 inhibition on tumor growth, metastasis, and response to treatment, providing essential data for the development of novel cancer therapies [59].

The molecular dynamic simulation, solvent-accessible surface area (SASA) analysis, and MMPB/GBSA analysis provided insightful data on the interactions between the HSP90AA1 target protein and various ligands, including cosmosiin, quercetin, rosmarinic acid, rubinin, and alvespimycin. Firstly, the RMSD analysis revealed stable receptor–ligand interactions throughout the simulation period, with RMSD values indicating minimal deviation from the initial structure. Notably, all systems maintained RMSD values below 3 Å, suggesting structural stability. However, subtle differences were observed in the mean RMSD values, with rubinin displaying the lowest mean deviation of 1.49 Å, indicating slightly tighter binding compared to other ligands. Secondly, RMSF analysis provided insights into the flexibility of receptor residues in the presence of ligands. While most residues exhibited average stability (<5 Å), notable variations were observed among the ligands. Rubinin exhibited the highest RMSF values, indicating greater flexibility, potentially due to loop pressure within the system. Furthermore, RoG analysis indicated consistent compactness of the systems over time, with minimal impact on ligand–receptor binding. However, slight differences were observed in maximum, mean, and minimum RoG values among the ligands, suggesting subtle variations in complex conformation. The SASA analysis revealed differences in the surface area of HSP90AA1 interacting with solvent molecules upon ligand binding. While all ligands displayed similar average SASA values, variations were observed in the lowest and highest SASA values, indicating distinct solvent accessibility patterns influenced by ligand structure. Finally, MMPB/GBSA analysis provided insights into the binding affinity and stability of the complexes. Highly negative net binding energies indicated strong intermolecular interactions and stable complexes for all ligands. Van der Waals forces predominantly contributed to complex stability, with electrostatic energies showing remarkable consistency across complexes.

As a result, this network-pharmacology-based investigation elaborates the mechanism of action of active drugs, their associated probable target genes, and link pathways to treat LC, laying the groundwork for additional experimental validation of the findings. Despite the fact that we have presented some intriguing data, more research and clinical studies are required to better investigate the potential of *C. myxa* and prove its medicinal potential.

## 5. Conclusions

This investigation elucidates the efficacy of multicomponent, multi-target drug combinations and uncovers novel therapeutic targets for LC treatment. Through the integration of network pharmacology, survival analysis, molecular docking, and molecular dynamics simulations, a comprehensive understanding of the molecular mechanisms underlying *C. myxa* in LC therapy has been achieved. The network analysis highlights the multi-targeting nature of *C. myxa* compounds, concurrently impacting multiple pathways implicated in LC progression. Particularly, the identification of the HSP90AA1 gene as a promising therapeutic target offers potential avenues for LC prevention and intervention, with the prospect of enhancing treatment efficacy. Despite these advancements, it is imperative to acknowledge the inherent limitations of the present study, underscoring the need for further phytochemical and pharmacological investigations to validate and expand upon our findings. This research establishes a robust scientific framework for exploring the therapeutic potential of *C. myxa* in LC management, paving the way for future studies aimed at optimizing its clinical application and improving patient outcomes.

## Figures and Tables

**Table 1:** Active constituents, their properties, and 2D structures.

Compound	MW	DL	OB	2D Structure	CID
Allantoin	158.12	0.88	0.55	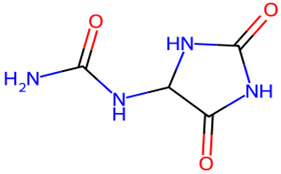	204
Beta-sitosterol	414.7	0.78	0.55	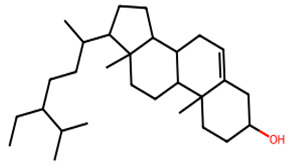	222,284
Catechin	290.27	0.64	0.55	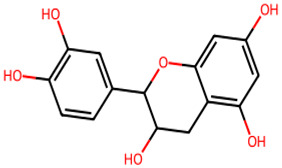	9064
Cosmosiin	432.4	0.59	0.55	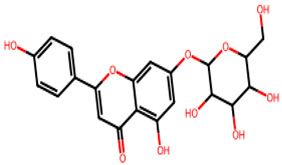	5,280,704
Gentisic acid	154.12	0.3	0.56	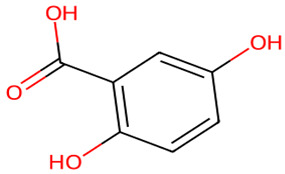	3469
Kaempferol	286.24	0.5	0.55	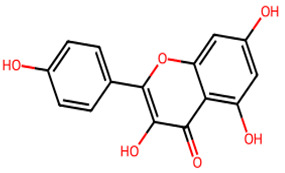	5,280,863
Quercetin	302.23	0.52	0.55	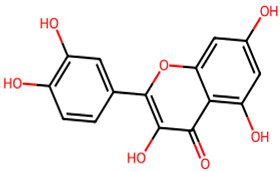	5,280,343
Rosmarinic acid	360.3	0.37	0.56	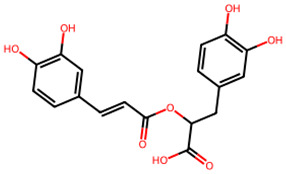	5,281,792
Rubinin	392.8	0.18	0.55	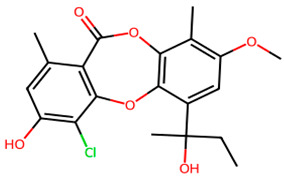	101,316,842
Stigmastanol	416.7	0.29	0.55	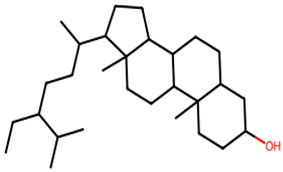	241,572

**Table 2:** Active constituents, their class, and CytoHubba’s different scoring algorithms.

Compound	Class	Degree	MNC	MCC	Closeness	Betweenness
Allantoin	Azoles	128	1	110	249.5	61,187.7
Beta-sitosterol	Steroids	134	1	109	248.833	57,693.4
Cosmosiin	Flavonoids	103	1	102	244.167	45,039
Catechin	Flavonoids	30	1	10	175.133	8527.81
Gentisic acid	Benzenoids	106	1	105	246.167	53,199
Kaempferol	Flavonoids	129	1	107	247.5	32,115.4
Quercetin	Flavonoids	113	1	108	246.85	33,069.9
Rosmarinic acid	Cinnamic acids	110	1	106	245.517	58,365.3
Rubinin	Flavonoids	100	1	100	242.833	56,869.3
Stigmastanol	Steroids	104	1	103	244.833	53,677.2

**Table 3:** Docking results of 10 active ingredients and one control drug with HSP90AA1.

Protein	Compound	Binding Affinity (kJ/mol)	RMSD (Å)	Interacting Residues
HSP90AA1	Cosmosiin	−7.3	1.183	ARG A:46, ASN A:51, ASP A:54
	Rosmarinic acid	−7.2	2.552	LYA A:58, GLY A:97, MET A:98, GLY A:137
	Quercetin	−6.7	1.136	ASN A:51, GLY A:97, NET A:98, LEU A:107, HIS A:154
	Rubinin	−6.7	2.778	ASN A:51, ALA A:55, LYS A:58, MET A:98
	(+)-Catechin	−6.6	2.775	LEU A:107, ILE A:110, ALA A:111, VAL A:136
	Kaempferol	−6.5	1.485	GLU A:47, SER A:50, ASP A:54, GLY A:132
	Stigmastanol	−6.4	1.368	ALA A:55, MET A:98, LEU A:107
	Beta-sitosterol	−6.4	1.477	ALA A:55, LYS A:58, MET A:98, LEU A:107
	Alvespimycin	−6.0	2.631	ASN A:51, ASP A:102, HIS A:154
	Allantoin	−5.5	2.967	ASN A:51, ASP A:54, THR A:184
	Gentisic acid	−4.8	0.884	LEU A:107, ALA A:111, VAL A:136, PHE A:138

**Table 4:** Docked complexes MMPB/GBSA energies in kcal/mol.

Parameter	HSP90AA1_Cosmosiin	HSP90AA1_Quercetin	HSP90AA1_Rosmarinic Acid	HSP90AA1_Alvespimycin	HSP90AA1_Rubinin
**MM/GBSA**					
Energy van der Waals	−41.25	−46.01	−44.69	−55.94	−51.01
Energy Electrostatic	−11.02	−10.67	−12.08	−13.71	−14.08
Total Gas Phase Energy	−52.27	−56.68	−56.77	−69.65	−65.09
Total Solvation Energy	8.08	7.14	7.78	8.09	8.66
Net Energy	−44.19	−49.54	−48.99	−61.56	−56.43
**MM/PBSA**					
Energy van der Waals	−41.25	−46.01	−44.69	−55.94	−51.01
Energy Electrostatic	−11.02	−10.67	−12.08	−13.71	−14.08
Total Gas Phase Energy	−52.27	−56.68	−56.77	−69.65	−65.09
Total Solvation Energy	7.52	8.06	7.65	8.04	8.16
Net Energy	−44.75	−48.62	−49.12	−61.61	56.93

## Data Availability

All the data are available publicly and within the article.
